# Supplementary material for: A survey of the views and capabilities of community pharmacists in Western Australia regarding the rescheduling of selected oral antibiotics in a framework of pharmacist prescribing
Source: PeerJ. 2018 May 7;6:e4726. doi: 10.7717/peerj.4726 (PMC5944433; doi:10.7717/peerj.4726)
Supplement: Supplemental Information 1 [file peerj-06-4726-s001.pdf]

QUESTIONNAIRE ON OVER-THE-COUNTER (OTC) PRESCRIBING OF ANTIBIOTICS FOR A LIMITTED RANGE OF INFECTIONS

Please place completed questionnaire in reply paid envelope provided and post back to School of Pharmacy, Curtin University

Section A: Participants' demographic information

Please provide us with the following information to assist with the analysis of data

1. Gender: ☐ F ☐ M
2. Age: ☐ 21-30 ☐ 31-40 ☐ 41-50 ☐ 51-60 ☐ 61+
3. How many years have you been registered as a pharmacist? ☐ 1-5 ☐ 6-20 ☐ 21+
4. How many years have you worked in a community pharmacy? ☐ 1-5 ☐ 6-20 ☐ 21+
5. Which of the following best describes the position(s) you hold in this community pharmacy? (tick more than one box if appropriate)

   ☐ Proprietor ☐ Manager ☐ Pharmacist- in charge ☐ Employed pharmacist
   ☐ Consultant pharmacist ☐ Locum pharmacist ☐ Other……………………………............ (Please specify)

Please tick the appropriate boxes that best correspond with your community pharmacy (where you work)

6. What is the location of this community pharmacy?

   ☐ Metropolitan community pharmacy (Go to question 8 below) ☐ Rural community pharmacy

7. If you selected rural community pharmacy above, please indicate which classification according to the PhARIA scale classifies your community pharmacy:

   ☐ 1 ☐ 2 ☐ 3 ☐ 4 ☐ 5 ☐ 6 ☐ Or please provide postcode of pharmacy………….......

8. Does this pharmacy have a confidential counselling room or a quiet area suitable for patient consultation and/or counselling?

   ☐ Yes ☐ No

9. Does this pharmacy have a forward dispensing area? (A forward dispensing area is where a patient sits at a desk or corral or similar with the pharmacist to discuss all their prescription issues)

   ☐ Yes ☐ No

10. Which of the following best describes the size of operation of this community pharmacy?

    ☐ Small (turnover ≤$1m) ☐ Medium (between $1m to $2m) ☐ Large (turnover >$2m)

11. Which of the following best describes the setting of the pharmacy?

    ☐ Strip shopping area or group of shops ☐ City centre
    ☐ Neighbourhood shopping centre ☐ Stand-alone
    ☐Situated next to doctor's surgery ☐ Regional shopping centre
    ☐ Medical centre ☐ Other (please state)……………………………………

Section B: Pharmacist's views on 'down-scheduling' of antibiotics

1. Currently a small selection of oral antifungal drugs (e.g. fluconazole) and antiviral drugs (e.g. famciclovir) are available over-the-counter (OTC). Please provide your views concerning OTC prescribing of some selected oral antibiotics that could be down scheduled to Schedule 3. Please indicate by ticking in the box that corresponds with how you consider the following statements.

	Statement	Strongly Agree	Agree	Neutral	Disagree	*Strongly disagree*
a)	*Selected oral antibiotics should be down scheduled from Schedule 4 to Schedule 3.*					
b)	*OTC prescribing of selected oral antibiotics offers better use of pharmacist's knowledge and skills.*					
c)	*The provision of selected OTC oral antibiotics increases the recognition of pharmacists' knowledge and skills by pharmacy clients.*					
d)	*The provision of OTC oral antibiotics helps ease the work load on doctors.*					
e)	*OTC prescribing of oral antibiotics is essential for a limited range of infections.*					
f)	*The capability to prescribe selected OTC oral antibiotics ensures that patients can be treated in a timely manner.*					
g)	*The availability of selected OTC oral antibiotics increases patient's easy access to antibiotics.*					
h)	*The current design of your pharmacy would allow the diagnosis of a limited range of infections and supply of oral antibiotics.*					
i)	*The provision of OTC oral antibiotics can potentially increase resistance to antibiotics.*					

2. If the following oral antibiotics were being considered for down scheduling (from Schedule 4 to Schedule 3 medicines), to what level would you be comfortable to prescribe the listed antibiotics OTC for appropriate indications: (Please tick the box that is most appropriate) *Please note: table continues on next page…*

Statement	Strongly Agree	Agree	Neutral	Disagree	*Strongly disagree*
Amoxicillin					
Phenoxymethylpenicillin					
Dicloxacillin					
Flucloxacillin					
Amoxicillin + clavulanic acid					
Cephalexin					
Cefuroxime					
Cefaclor					
Roxithromycin					
Azithromycin					
Clarithromycin					
Erythromycin					
Metronidazole					

Statement	Strongly Agree	Agree	Neutral	Disagree	Strongly disagree
Trimethoprim					
Norfloxacin					
Ciprofloxacin					
Rifampicin					
Doxycycline					
Nitrofurantoin					

3. If you were able to prescribe oral antibiotics OTC, how many patients per week on average do you estimate, would seek your advice for conditions where you would prescribe antibiotics?

None	1-3	4-6	7-9	>10
☐	☐	☐	☐	☐

Section C: Vignettes

The vignettes are simulations of real scenarios used to describe particular events or situations in order to elicit participant knowledge, attitudes and opinions based on responses in depicted pre-constructed case studies.

The following 7 vignettes are related to patients with infections where oral antibiotics registered in Australia could be prescribed. Responses to vignettes are based on case scenarios where ALL oral antibiotics for this exercise are down scheduled to Over-The-Counter (OTC) in Australia, and **you are the pharmacist assessing the patient to prescribe suitable oral antibiotic(s) where appropriate**. Please assume that the case study patients do not have any known allergies to antibiotics and that the infections are initial presentations unless specified otherwise in the case.

Case 1: Otitis media

Presenting symptom(s): A 5 year old child is brought to your pharmacy by her mother with a history of signs and symptoms suggestive of ear infection. As the pharmacist, based on the information you have gathered, you are satisfied that the child has mild otitis media and you now need to determine appropriate treatment.

Please select below the treatment you would prescribe for this patient

☐ Amoxicillin 15mg/kg up to 500mg orally, 8 hourly for 5 days
☐ Cefuroxime 15mg/kg up to 500mg orally, 12 hourly for 5 days
☐ Cefaclor 10mg/kg up to 500mg orally, 8 hourly for 5 days
☐ Erythromycin 10 mg/kg (up to 500 mg) orally, every 6 hours for 5 days
☐ Cephalexin 12.5 mg/kg (maximum 500 mg) orally, every 6 hours for 5 days.
☐ Other, (please specify regimen*):* ____________________
☐ I would refer patient to GP straight away *(please go to case 2)*

After 3 days of the treatment prescribed above, the patient comes back to your pharmacy with no symptomatic improvement. Please indicate what follow up and advice you would provide for this patient

☐ Select another antibiotic(s), *(please name):*____________________
☐ Increase dose of antibiotic(s)
☐ Consider alternative diagnosis and treat accordingly
☐ Refer patient to GP
☐ Other, *(please specify):* ____________________

Case 2 and 3 on the next page…

Case 2: UTI in pregnancy

Presenting symptom(s): A 26 year old 24 weeks pregnant woman presents with burning pain on urination since yesterday. After further questioning, based on the information you have gathered, you are satisfied that she has a mild urinary tract infection.

Please select below the treatment you would prescribe for this patient

☐ Amoxicillin 875mg + clavulanic acid 125mg orally, 12 hourly for 10 days
☐ Cephalexin 500mg orally, 6 hourly for 10 days
☐ Nitrofurantoin 100mg orally, 12 hourly for 5 days
☐ Trimethoprim 300mg orally, daily for 10 days
☐ Metronidazole orally, 200–400 mg every 8–12 hours, up to 4 g daily
☐ Other, *(please specify regimen):*____
☐ I would refer patient to GP straight away *(please go to case 3)*

After 3 days of the treatment prescribed above, the patient comes back to your pharmacy with no symptomatic improvement. Please indicate what follow up and advice you would provide for this patient

☐ Select another antibiotic(s), *(please name)*:__________________________________
☐ Increase dose of antibiotic(s)
☐ Consider alternative diagnosis and treat accordingly
☐ Refer patient to GP
☐ Other, *(please specify)*:______________________________________

Case 3: Community acquired pneumonia (CAP) with acute respiratory symptoms

Presenting symptom(s): A 30 year old male presents at your pharmacy with a history of acute cough, sputum production, chest pain and fever over the past 24 hours. After further questioning, you are satisfied that the patient has a mild CAP.

Please select below the treatment you would prescribe for this patient

☐ Amoxicillin 1g orally, 8 hourly for 5-7 days
☐ Doxycycline 200mg orally, for the first dose, then 100mg daily for a further 5 days
☐ Clarithromycin 250mg orally, for the first dose, then 100mg daily for a further 5-7 days
☐ Azithromycin 500 mg orally once on day 1, followed by 500 mg once daily for 2 days or 250 mg once daily for 4 days.
☐ Cephalexin 500mg orally, 6 hourly for 7-10 days
☐ Ciprofloxacin 500mg orally, 12 hourly for 10 days
☐ Other, *(please specify regimen):*____
☐ I would refer patient to GP straight away (please go to case 4)

After 24 hours of the treatment prescribed above, the patient comes back to your pharmacy with no symptomatic improvement. Please indicate what follow up and advice you would provide for this patient

☐ Select another antibiotic(s), please name: __________________________________
☐ Increase dose of antibiotic(s)
☐ Consider alternative diagnosis and treat accordingly
☐ Refer patient to GP
☐ Other, *(please specify):* ______________________________________

Case 4 and 5 on the next page…

Case 4: Tonsillitis

Presenting symptom(s): A 10 year old girl presents at your pharmacy with tenderness and swelling in the throat area, fever of 39ºC, and tonsillar exudate and no cough. After further questioning, you are satisfied that the symptoms are consistent with bacterial tonsillitis.

Please select below the treatment you would prescribe for this patient

☐ Phenoxymethylpenicillin 15mg/kg up to 500mg orally, 12 hourly for 10 days
☐ Erythromycin 250–500 mg orally, every 6–8 hours.
☐ Doxycycline orally, 200 mg on day 1 (as a single dose or 100 mg twice daily), then 100 mg once daily.
☐ Amoxicillin 1g orally, 8 hourly for 5-7 days
☐ Other, *(please specify regimen):*____
☐ I would refer patient to GP straight away (please go to case 5)

After 3 days of the treatment prescribed above, the patient comes back to your pharmacy with no symptomatic improvement. Please indicate what follow up and advice you would provide for this patient

☐ Select another antibiotic(s), *(please name)*:____
☐ Increase dose of antibiotic(s)
☐ Consider alternative diagnosis and treat accordingly
☐ Refer patient to GP
☐ Other, *(please specify):*____

Case 5: Chlamydial urethritis with frequency, dysuria and discharge

Presenting symptom(s): A 28 year old male who has been treated for chlamydial urethritis in the past presents at your pharmacy with a history of frequency, dysuria and discharge. On questioning he indicated he had unprotected sex 5 days earlier. He states he has been treated previously with prescribed antibiotic(s) for his condition.

Please select below the treatment you would prescribe for this patient

☐ Azithromycin 1g orally, as a single dose
☐ Azithromycin 500mg orally, on day 1, followed by 250mg orally, on day 2-5
☐ Doxycycline 100mg orally, 12 hourly for 7 days
☐ Erythromycin 250–500 orally, mg every 6–8 hours.
☐ Amoxicillin 1g orally, 8 hourly for 5-7 days
☐ Other, *(please specify regimen)*:____
☐ I would refer to GP straight away *(please go to case 6)*

After 3 days of the treatment prescribed above, the patient comes back to your pharmacy with no symptomatic improvement. Please indicate what follow up and advice you would provide for this patient

☐ Select another antibiotic(s), please name: ____
☐ Increase dose of antibiotic(s)
☐ Consider alternative diagnosis and treat accordingly
☐ Refer patient to GP
☐ Other, *(please specify):*____

Case 6 and 7 on the next page…

Case 6: Mild early cellulitis

Presenting symptom(s): A 60 year old male patient presents at your pharmacy with a 3 day history of painful, red and hot swelling on the left lower leg. After further questioning, you are satisfied that this is cellulitis. The patient has no past medical history of chronic conditions and is not on any medications.

Please select below the treatment you would prescribe for this patient

☐ Dicloxacillin 500mg orally, 6 hourly for 7-10 days
☐ Flucloxacillin 500mg orally, 6 hourly for 7-10 days
☐ Phenoxymethylpenicillin 500mg orally, 6 hourly for 10 days
☐ Cephalexin 500mg orally, 6 hourly for 7-10 days
☐ Doxycycline 100mg orally, 12 hourly for 7 days
☐ Erythromycin 250–500 mg orally every 6–8 hours.
☐ Other, *(please specify regimen):*____
☐ I would refer to GP straight away (please go to case 7)

After 3 days of the treatment prescribed above, the patient comes back to your pharmacy with no symptomatic improvement. Please indicate what follow up and advice you would provide for this patient

☐ Select another antibiotic(s), *(please name):* ____
☐ Increase dose of antibiotic(s)
☐ Consider alternative diagnosis and treat accordingly
☐ Refer patient to GP
☐ Other, *(please specify):* ____

Case 7: Acute pyelonephritis

Presenting symptom(s): A 38 year old woman presents with low grade fever and lower back pain for several days but no nausea or vomiting. She has had burning urine for several days and Ural® sachets has not alleviated the condition. She has had similar symptoms in the past. As the pharmacist, you conclude the patient has acute pyelonephritis.

Please select below the treatment you would prescribe for this patient

☐ Amoxicillin 875mg + clavulanic acid 125mg orally, 12 hourly for 10 days
☐ Cephalexin 500mg orally, 6 hourly for 10 days
☐ Trimethoprim 300mg orally, daily for 10 days
☐ Azithromycin 500 mg once orally on day 1. Then give 500 mg once daily for 2 days or 250 mg once daily for 4 days.
☐ Doxycycline 100mg orally, 12 hourly for 7 days
☐ Other, *(please specify regimen)*:____
☐ I would refer patient to GP straight away

After 3 days of the treatment prescribed above, the patient comes back to your pharmacy with no symptomatic improvement. Please indicate what follow up and advice you would provide for this patient

☐ Select another antibiotic(s), *(please name)*:____
☐ Increase dose of antibiotic(s)
☐ Consider alternative diagnosis and treat accordingly
☐ Refer patient to GP
☐ Other, *(please specify):*____

The end: Thank you very much for your time.

Please place completed questionnaire in reply paid envelope provided and post back to School of Pharmacy, Curtin University.
